# An Easy Approach to Calculating Estimated Energy Requirements

**Published:** 2006-09-15

**Authors:** Shirley Gerrior, WenYen Juan, Basiotis Peter

**Affiliations:** Cooperative State Research Education and Extension Service, U.S. Department of Agriculture; Center for Nutrition Policy and Promotion, U.S. Department of Agriculture, Washington, DC; Center for Nutrition Policy and Promotion, U.S. Department of Agriculture, Washington, DC

## Abstract

The Dietary Reference Intakes (DRIs) define the daily requirement for energy as the Estimated Energy Requirement (EER). The EER is based on calculations that account for an individual's energy intake, energy expenditure, age, sex, weight, height, and physical activity level. Including physical activity level in the calculations makes determining energy expenditure possible and achieving energy balance a more realistic goal. However, physical activity level is often difficult to measure and accurate assessment of energy expenditure not always possible. We provide an easy way to calculate daily EERs for adults based on physical activity level. We use the EER equations of the DRI Committee and provide a spreadsheet template for the calculation of physical activity level. This technique accounts for all factors and measurements to determine physical activity level and energy expended from daily physical activity. It should prove a useful approach in research, clinical, and public health settings.

## The Estimated Energy Requirement

In the report *Dietary Reference Intakes for Energy, Carbohydrate, Fiber, Fat, Fatty Acids, Cholesterol, Protein and Amino Acids (Macronutrients)* (1), the Institute of Medicine (IOM) of The National Academies sets the Dietary Reference Intake for energy as the Estimated Energy Requirement (EER). The motivation for establishing the EER was tied to public health awareness and concern about the increased prevalence of overweight and obesity in North America and the need to adequately assess energy balance (2). The EER is calculated from a set of equations and factors that account for an individual's energy intake, energy expenditure, age, sex, weight, height, and physical activity level (PAL). Based on the energy intake needed to maintain energy balance in individuals with healthy weights, the EER consists of predictive equations for calculating the amount of energy intake that will maintain any individual's body weight, as measured by doubly labeled water studies (1).

The equations used to calculate the EER consider factors that affect an individual's daily energy expenditure, account for increments in energy expenditure based on physical activity, and quantify the effect of the PAL on total energy expenditure (TEE) (1). All aspects of energy expenditure in a 24-hour period, including energy expended in synthesizing new tissues or as a result of lactation or growth and development, are accounted for (3). This approach differs from the more commonly used predictive equations used in clinical settings (e.g., the Harris–Benedict equation) to estimate basal energy expenditure or TEE or to assess energy requirement (4). In addition, it has the potential for use in clinical settings as well as academic and research settings for nutritional and physical activity assessment and for weight management studies when energy expenditure from physical activity is not measurable. But understanding EER equation components and factors can be time consuming.

Using the EER predictive equations, we devised an easy way to calculate daily energy requirements for both men and women based on PAL. We have created a template using a spreadsheet to simplify the EER calculation process. The template includes components of energy expenditure that include PAL calculation in user-friendly spreadsheets. This technique accounts for all factors and measurements to determine PAL and energy expended from daily physical activity and should provide a useful approach for academic, research, clinical, and public health settings.

## EER Components and Energy Balance

Components of the EER are incorporated in the template spreadsheet using Microsoft Excel 2000 (Microsoft Corp, Redmond, Wash) (Table) to calculate TEE based on PAL for a man or a woman aged 19 years or older. The basic and largest component of the TEE is basal energy expenditure (BEE). BEE reflects the basic metabolic rate (BMR), or the daily energy needed to sustain cell metabolism and associated life processes, extrapolated to a 24-hour period. The BEE is positively correlated with body size and composition and is easily predicted from age, sex, and height, but it is unclear how it may be affected by physical activity (5,6). Another component, the thermic effect of food (TEF) and thermoregulation, has a smaller impact on TEE. Contributed by digestive and neural processes associated with eating, the TEF increases energy metabolism and accounts for about 8% to 10% of an individual's daily energy expenditure, but any effect of physical activity or exercise on TEF is likely to be fairly small (5). Energy expended from physical activity is the most variable part of the TEE and is of major importance to achieving energy balance. Depending on how much an individual's physical activity varies from day to day, it can be very low or high, but it usually accounts for approximately 20% to 30% of energy expended (1,6,7). To account for this variability, the EER equations include a physical activity coefficient (PA). This coefficient considers the impact of the duration and intensity of the physical activity performed and the efficiency of performance. An excess postexercise oxygen consumption (EPOC) factor, estimated at about 15%, is also included to adjust for an additional increase in energy expenditure induced for a period of time after the completion of a bout of physical activity (1).

### Physical activity: metabolic equivalents and PAL

Doubly labeled water data of both predicted and observed levels of physical activity were used by the IOM to assess physical activity patterns and energy expenditure of individuals for placement into one of four PAL categories: sedentary, low active, active, or very active (1). In the EER equations, the PAL categories are closely linked to energy expended during physical activity in terms of metabolic equivalents (METs). A MET is a numerical value that represents a multiple of the resting metabolic rate for a particular activity. This value applies to the level of energy expenditure achieved during the performance of a specific activity at a designated intensity and provides a way of expressing the total caloric cost of the activity. One MET equates to a rate of O_2_ consumption of 3.5 milliliters per kilogram of body weight per minute (ml/kg/min) in adults. MET values between 1.0 and 12.0 represent the typical range of PAL, from light to moderate to vigorous. The MET value applies to the level of energy expenditure achieved during the performance of an activity and provides a way of expressing the total caloric cost of the activity (Δ PAL).

The PAL provides information about the duration and intensity of a set of different activities performed during a 24-hour period and the relative differences in usual levels of physical activity. The relationship between METs and PAL is incorporated into the sample EER template spreadsheet (Table) such that each physical activity performed in a 24-hour period is assigned a MET value based on its intensity and duration. A resulting set of MET values is subsequently used to calculate the PAL for determination of the PA needed to estimate the TEE.

## Template Components and Use

This template identifies the fields needed for data entry by the user and inserts factors for the automatic calculation of previously defined energy components. Data entry fields required are an individual's age (in years), weight (in kilograms), height (in meters), a list of activities performed and their intensity (METs), and the duration (minutes) of each in the past 24 hours. Factors inserted into the spreadsheet are the EPOC factor of 1.15, the TEF factor of 0.9, and a standard factor to calculate the PAL of 1 MET (or 0.0175 kcal per kg) per minute of activity (1). In addition, we created several other factors that are transparent to the user for the simple translation of an individual's reported physical activity into energy expenditure. To demonstrate, we use a reference active man of 30 years, 1.77 meters, and 70 kg and a reference active woman of 30 years, 1.63 meters, and 54 kg in the Table.

### How the calculation works

After the required data fields are entered, the template spreadsheet automatically calculates the BEE written as follows:

**For men:**

BEE = 293 − 3.8 × age (years) + 456.4 × height (meters) + 10.12 × weight (kg)

**For women:**

BEE = 247 − 2.67 × age (years) + 401.5 × height (meters) + 8.6 × weight (kg)

The next step is the automatic calculation of the impact of each reported physical activity on energy expenditure (Δ PAL). This formula, set in the template, is as follows:

**Table:** 

Δ PAL =	(METs − 1) × [(1.15/0.9) × Duration (minutes)]/1440)

	BEE/[0.0175 × 1440 × weight (kg)]

After the Δ PAL is calculated for each physical activity, the physical activity category (PAL: sedentary, low active, active, or very active) is determined based on the basal activity impact on energy expenditure (a factor of 1.1) and the sum of all activities (sum of Δ PAL). This factor accounts for TEF and postexercise increase in energy expenditure. The PAL is automatically calculated as PAL = 1.1 + sum of Δ PALi, where Δ PALi is the list of each reported activity impact on energy expenditure.

The PAL is automatically calculated from the sum of Δ PALi and used to determine the PA based on the following criteria:

**For men:**

Sedentary: PA = 1.0, when 1.0 ≤ PAL <1.4

Low active: PA = 1.12, when 1.4 ≤ PAL <1.6

Active: PA = 1.27, when 1.6 ≤ PAL <1.9

Very active: PA = 1.54, when 1.9 ≤ PAL <2.5

**For women:**

Sedentary: PA = 1.0, when 1.0 ≤ PAL <1.4

Low active: PA = 1.14, when 1.4 ≤ PAL <1.6

Active: PA = 1.27, when 1.6 ≤ PAL <1.9

Very active: PA = 1.45, when 1.9 ≤ PAL <2.5

The formula to determine the PA for men using the Microsoft Excel logic function is:

IF(PAL ≥1.9, "1.54", IF(PAL≥1.6,"1.27", IF(PAL≥1.4,"1.12", IF(PAL≥1,"1", ""))))

The formula to determine the PA for women using the Microsoft Excel logic function is:

IF(PAL ≥1.9, "1.45", IF(PAL≥1.6,"1.27", IF(PAL≥1.4,"1.14", IF(PAL≥1,"1", ""))))

After the template spreadsheet determines the PA, it is used to automatically calculate the TEE as:

**For men:**

TEE = 864 − 9.72 × age (years) + PA × [(14.2 × weight (kg) + 503 × height (meters)]

**For women:**

TEE = 387 − 7.31 × age (years) + PA × [(10.9 × weight (kg) + 660.7 × height (meters)]

## Considerations for Using the Template

Use of this template is not limited to active individuals; it can also be applied to individuals who have sedentary, low active, or very active lifestyles. The METs (entered by the spreadsheet user) for each of the physical activities performed will automatically calculate the PAL category necessary to determine the PA and the TEE. Nor is use of the template limited to Microsoft Excel format. The set of equations provided in the section "How the calculation works" can be applied to other statistical software programs (e.g., SAS) for faster data entry or analyses of large data sets.

Inherent in the EER predictive equations are some limitations that may affect template spreadsheet calculations and results for PAL. Using either the METs or PAL to calculate energy costs of physical activity may introduce some degree of error. The impact of METs on the calculated TEE and an individual's EER could result in an underestimate of energy costs for some activities and an overestimate for others compared with actual energy expenditure measurement (8). Likewise, for the PAL there may be differences associated with the increments in energy expenditure associated with physical activity. For most physical activities, these increments are directly proportional to body weight; however, when using the BEE, these increments are proportional to body weight multiplied by 0.75, resulting in less energy associated with these increments (3). The calculation of the PAL and the resulting PA coefficient is truncated at the very active level and thus does not account for more calories expended and required by exceeding this level of physical activity. This underestimates energy needs for people who regularly perform vigorous or very vigorous intensity activity for the recommended duration or longer.

## Implications for Practice

The template spreadsheet provides a useful tool and an easy way to calculate TEE and estimated energy requirements for adult men and women based on physical activity level. This approach estimates an individual's EER, thereby making energy balance a more attainable goal. By incorporating the most current dietary recommendations for energy requirements in terms of an individual's physical activity level, this approach provides a useful way to manage weight and to assess physical activity as part of a healthy and active lifestyle. Most recently this approach has been used in the MyPyramid Tracker to integrate energy intake from food with energy expenditure from physical activity to determine energy balance status. More information about the United States Department of Agriculture's food guidance system and this useful tool is available from www.MyPyamidTracker.gov.

## Figures and Tables

**Table:** Template for Calculation of Estimated Energy Requirement

This table is available as a Microsoft Excel spreadsheet to enable users to calculate their EER based on PAL. You must have Microsoft Excel to use this table.	**Download the Excel spreadsheet** (XLS 17k)
